# During *Aspergillus nidulans* nitrogen-limited biofilm formation, mitophagy is independent of mitochondrial fission

**DOI:** 10.1080/27694127.2025.2547194

**Published:** 2025-08-22

**Authors:** Hari Krishnan Balasubramanian, Stephen A. Osmani

**Affiliations:** a Molecular, Cellular, and Developmental Biology Program, Ohio State University, Columbus, USA, OH; b Molecular, Cellular, and Developmental Biology Program, Ohio State University, Columbus, USA, OH

**Keywords:** *Aspergillus nidulans*, autophagy, biofilm formation, carbon, mitochondrial fission, mitophagy, nitrogen, redox regulation

## Abstract

During chronic infections, biofilms are resistant to both antimicrobial agents as well as the host immune system, often giving rise to recalcitrant persister cells with reduced mitochondrial function rendering biofilm infections difficult to cure. How mitochondrial dynamics and fate are regulated during fungal biofilm formation is poorly understood. In this study, we used live cell microscopy to track mitochondrial morphology during *Aspergillus nidulans* in vitro biofilm formation. We show that mitochondria undergo fragmentation during early biofilm development, and that externally induced oxidative stress similarly induces mitochondrial fragmentation, indicating a role for redox regulation in this process. Deletion of core components of the mitochondrial fission machinery resulted in a swollen mitochondrial phenotype. Mitochondria in the fission-mutant strains are known not to complete fragmentation in response to externally induced oxidative stress, and we show that this results in a “beads on a string” phenotype. We further show that mitochondria remain unfragmented during biofilm formation in the fission-mutant strains, although other biofilm cellular modifications, like disassembly of microtubules, are unaffected. We report that mitophagy is triggered during biofilm development in nitrogen-limiting conditions independently of mitochondrial fission. This indicates mitochondrial fission is dispensable for mitophagy during biofilm development with limiting nitrogen. We further note that general autophagy, but notably not mitophagy, is triggered during biofilm development in carbon-limiting conditions, demonstrating differential regulation of mitochondrial fate in response to specific nutritional limitations during fungal biofilm formation.

## Introduction

Biofilms are complex macroscopic colonies formed by microbes that adhere to a surface and grow as a complex community. In nature, filamentous fungi can grow as biofilms with increased tolerance against ecological stresses. Biofilms are characterized by the presence of an extracellular matrix comprising proteins, polysaccharides, lipids, and nucleic acids^[1–3]^. Pathological fungal biofilms are resistant to both antifungal agents as well as the host immune system, forming persistent sources for infection cycles. Fungal biofilms forming on the abiotic surfaces of implanted medical devices like heart valves, coronary stents, cochlear implants, etc., are extremely hard to treat and can cause life-threatening complications in patients with contaminated implants^[4,5]^. Additionally, the biofilm mode of growth has important industrial applications owing to significantly higher yields of commercially important products like enzymes, organic acids, secondary metabolites like statins, etc. that can be obtained from biofilm cultures^[6–8]^. However, the cell biology specific to fungal biofilm cells is not well understood.

The heterogeneity in microenvironments within a biofilm elicits distinct responses from cells within different regions of a biofilm. This results in significantly different biological processes in biofilm cells as compared to unattached free-growing planktonic cells, including elevated drug efflux activity^[9]^ and altered metabolic states^[10,11]^. Studies have shown biofilm cells to possess up to 100× higher drug resistance than their free-growing planktonic counterparts^[12–14]^, and resistance to drugs in biofilm cells was found to increase as the biofilms develop and mature^[15,16]^.

Molecular oxygen (O_2_) acts as the terminal electron acceptor for aerobic mitochondrial respiration in most eukaryotes including fungi and is also critical for the biosynthesis of vital compounds including sterols, fatty acids, and porphyrins^[17–19]^. Biofilm formation is associated with the generation of intrinsic hypoxic microenvironments, and studies have reported the development of an O_2_ gradient within biofilms as they grow and undergo maturation^[20,21]^. Our lab has previously reported multiple cellular biological modifications occurring during biofilm formation in the founder biofilm cells, including disassembly of microtubules (MTs)^[22]^, modifications of the Golgi apparatus and endoplasmic reticulum (ER), and dispersal of endocytic actin patches^[23]^. Importantly, these modifications were found to be under the control of the hypoxic transcription factor SrbA^[22,23]^, suggesting that these modifications occur in response to self-generated biofilm hypoxia. Supporting this idea, it was also shown that allowing increased air exchange above static liquid biofilm cultures promoted reversal of MT disassembly in early-stage biofilms^[22]^. As the lack of O_2_ results in the inability to efficiently generate cellular energy through aerobic mitochondrial respiration, it is proposed that these energy conserving modifications enable hypoxia adaptation and survival after entering a state of growth arrest and dormancy.

As the primary O_2_ consumers in eukaryotic cells, mitochondria are highly susceptible to fluctuations in O_2_ availability, yet not much is known about how mitochondrial dynamics and fate are regulated under O_2_ depletion during fungal biofilm formation. Fungal biofilms are known to give rise to drug resistant recalcitrant cells called persister cells^[24,25]^, a spontaneously and stochastically generated sub-population of genetically identical clonal cells with phenotypic variations. Proteomic analysis of *Candida albicans* biofilm persister cells has revealed a marked downregulation of key enzymes involved in glycolysis, the tricarboxylic acid (TCA) cycle, and protein synthesis^[25]^. This suggests significantly reduced mitochondrial function in these cells, yet there are limited studies on how the mitochondrial morphology and fate are regulated during fungal biofilm formation.

*Aspergillus nidulans* is a multicellular eukaryotic filamentous ascomycete with elongated multinucleated hyphal cells that are ideal for live cell microscopy. Using genetic tools and live cell microscopy, we tracked mitochondrial morphology during biofilm formation in biofilm founder cells. The results of this study demonstrate that mitochondria undergo fragmentation during early biofilm formation, and that this process relies on the mitochondrial fission machinery. We find that the loss of mitochondrial fragmentation during early biofilm formation does not seem to impair other cellular modifications occurring at this stage. However, we do find that impairing mitochondrial fission results in a swollen mitochondrial phenotype. We also report the appearance of a “beads on a string” mitochondrial morphology when the mitochondrial fission-mutant cells are exposed to induced oxidative stress and discuss the potential mechanism giving rise to such a phenotype. We find that during biofilm formation in nitrogen-limiting conditions, mitophagy is induced in founder biofilm cells but not in carbon-limiting conditions and discuss the potential mechanism leading to this difference. We find that the loss of biofilm mitochondrial fragmentation does not impact the induction of mitophagy during biofilm formation in nitrogen-limiting conditions and discuss the potential cause for mitochondrial fission being unessential for mitophagy during biofilm formation in nitrogen-limiting conditions.

## Materials and methods

### General techniques and strain generation

Table S1 lists the *A. nidulans* strains used in this work. The strains were generated using classical techniques including transformation of gene-targeting constructs generated via fusion PCR and also by genetic crosses^[26–29]^. Gene deletion and/or tagging were carried out at their endogenous locus in a *kuA* deleted background, and the *kuA* deletion was eliminated subsequently via genetic crosses. Diagnostic PCR using primers flanking the targeting construct was carried out to verify the homologous integration of deletion/tagging constructs.

### Cell culture for microscopy

Fresh *A. nidulans* spores were harvested from rich media agar-overlay plates incubated at 32°C for 36–40 h. The spores were washed twice in 0.2% Tween-80 and stored at 4°C in spore stock solution (0.02% Tween-80 plus 0.85% NaCl). 35 mm glass-bottomed dishes (MatTek) containing 3 ml minimal medium were used for growth of cells and biofilm formation for live-cell microscopy. Spores were inoculated at a concentration of 2.5 × 10^5^ spores/ml for live-cell microscopy. Imaging media contained carbon (55.55 mM glucose), nitrogen (10 mM ammonium tartrate or urea), trace elements, MgSO_4_ (2 mM), KCl (7 mM), and phosphate buffer (6 mM KH_2_PO_4_ and 6 mM K_2_HPO_4_.3 H_2_O, pH adjusted to 6.5). For biofilm growth in carbon-limiting conditions, the carbon source was adjusted to 0.55 mM glucose. For biofilm growth in nitrogen-limiting conditions, the nitrogen source was adjusted to 0.5 mM ammonium tartrate. After inoculation of spores, imaging dishes were incubated at 25°C for 18 h for imaging cells at early growth stages and for longer at room temperature to monitor biofilm formation. For H_2_O_2_ treatments, 1 ml of imaging media was removed from the imaging dish into a 1.5 ml tube and the appropriate volume of H_2_O_2_ added and mixed thoroughly by pipetting before mixing this media back into the imaging dish.

### Microscopy, image-processing, and analysis

All imaging experiments were carried out on an UltraVIEW Vox CSUX1 spinning-disc confocal system (PerkinElmer). Images were captured with 9 to 14 z-slices using 0.8 µm z-spacing, and all images presented are maximum intensity projections unless specified otherwise. Images were processed using ImageJ software^[30]^.

To quantify mitochondrial width in selected sections, a linear ROI was drawn across the mitochondria, and the signal intensity was measured across the ROI to determine the distance between the two peaks representing the mitochondrial membrane on either side.

### Biofilm biomass analysis

To measure biofilm maturation, biofilms were allowed to develop inside the imaging dishes for 5 days and their total dry weights were measured. Briefly, biofilms were harvested from the dishes, followed by removal of media by blotting between paper towels and desiccating at 50°C for 3 days in a drying oven before measuring the dry weight.

## Results

### Mitochondria undergo fragmentation during A. nidulans biofilm formation

To investigate if mitochondrial morphology is modified during biofilm formation, we imaged mitochondria in pre-biofilm cells (Day 1) and biofilm cells (Day 3) tracking the mitochondrial outer membrane marker Tom20-GFP. Tom20 is a component of the mitochondrial outer membrane translocase (TOM) complex, which plays a central role in the import of mitochondrial proteins^[31]^. Tom20 has previously been fluorescently tagged and demonstrated to locate to the mitochondrial outer membrane in *A. nidulans*^[32]^.

**Figure 1.**
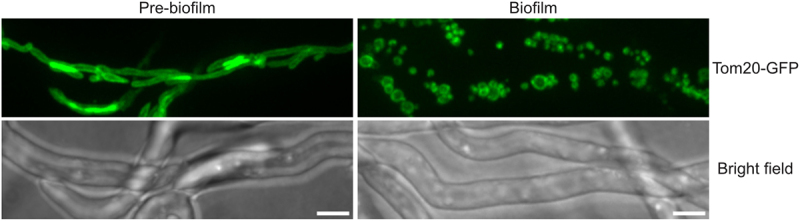
Mitochondria undergo fragmentation during *A. nidulans* biofilm formation. Spores from a strain expressing the mitochondrial outer membrane marker Tom20-GFP were inoculated in standard minimal media and allowed to grow at room temperature. Mitochondrial morphology was recorded before (Day 1) and during biofilm formation (Day 3) using spinning-disc confocal microscopy. White bars = 5 μm.

In pre-biofilm cells, mitochondria exist in a long tubular form characterized by apparent parallel tracks of the Tom20-GFP signal representing the mitochondrial outer membrane (Figure 1, Pre-biofilm). In all the biofilm cells imaged, mitochondria were found to be variably sized circular fragments, suggesting mitochondria had undergone extensive fragmentation during biofilm formation (Figure 1, Biofilm). Mitochondria are dynamic organelles undergoing constant fusion and fission events, and it is the balance between these that determines the mitochondrial morphology at any given time. The findings suggest that as the biofilm develops, founder biofilm cells undergo a shift in mitochondrial dynamics toward fission, resulting in mitochondrial fragmentation.

### Deletion of core components of the mitochondrial fission machinery causes mitochondria to swell in diameter

The core mitochondrial fission machinery in budding yeast comprises the dynamin-related GTPase Dnm1^[33,34]^, the outer membrane-anchored receptor Fis1^[35]^, and the adaptor protein Mdv1^[36]^. To determine if mitochondrial fragmentation during *A. nidulans* biofilm formation is similarly dependent on this mitochondrial fission machinery, we deleted these core components, namely DnmA, FisA, and Mdv1. Garrido-Bazán et al. previously identified AN8874 and AN6225 as DnmA and FisA respectively in *A*. *nidulans*^[37]^. Using the protein sequence of budding yeast Mdv1, we identified its homolog in *A*. *nidulans* as AN6867 based on reciprocal best hits BLAST analysis in FungiDB (https://fungidb.org/fungidb/app).

Garrido-Bazán et al. previously reported that the deletion of core components of the mitochondrial fission machinery (Δ*dnmA* and Δ*fisA*) results in growth inhibition and reduced conidiation^[37]^. We observed similar growth inhibition and reduced conidiation in all three mitochondrial fission-mutants, namely Δ*dnmA*, Δ*fisA*, and Δ*mdv1* (Figure S1). The similar phenotypes observed for the three mutants suggest they function in the same pathway and are essential for normal growth and asexual development in *A*. *nidulans*. Similar results have previously been reported in *A. fumigatus*, a closely related opportunistic pathogenic model, where deletion of Dnm1, Fis1 or Mdv1 resulted in growth inhibition, temperature sensitivity and reduced conidiation^[38]^. In another study, deletion of DnmA in *A. nidulans* was reported to result in growth inhibition when grown on agar plates but not, for unexplained reasons, when grown in submerged cultures^[39]^. We report similar findings, wherein the mitochondrial fission-mutants grow similarly to the wild-type (WT) strain when grown as submerged biofilms (Figure 4(C); see below) despite exhibiting growth inhibition on agar plates (Figure S1).

**Figure 2.**
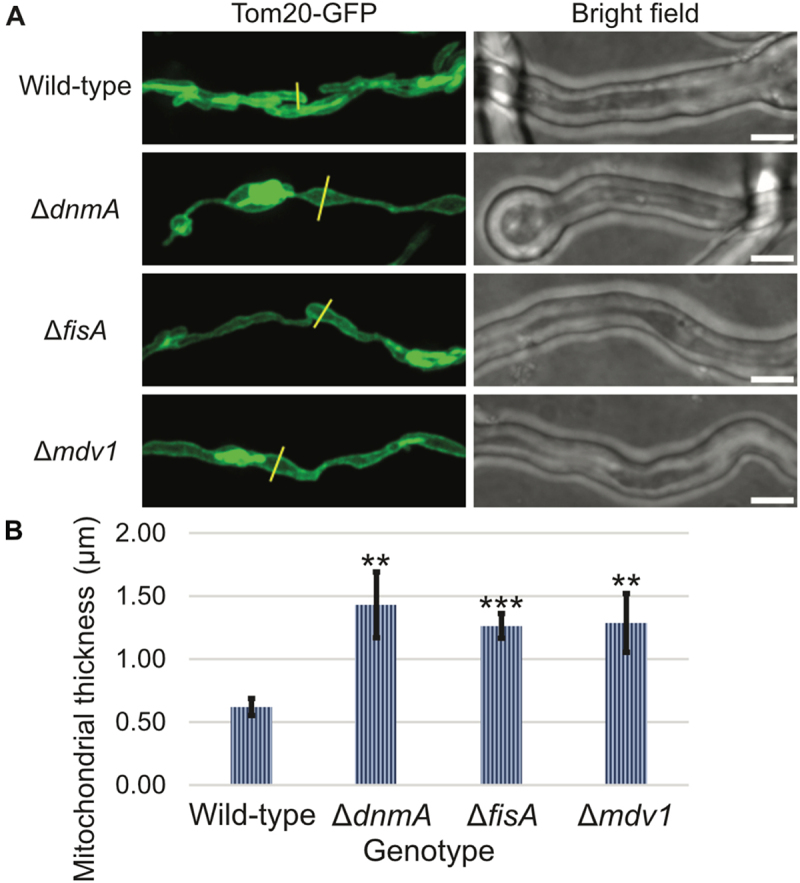
Deletion of core components of the *A. nidulans* mitochondrial fission machinery results in swollen-mitochondrial phenotype. (A) Spores from wild-type as well as the mitochondrial fission mutants were inoculated in minimal media and allowed to grow at 25°C for 18 h. Mitochondrial morphology was recorded using spinning-disc confocal microscopy. The yellow lines represent a sample of the regions (ROIs) chosen for measuring mitochondrial thickness. (B) Mitochondrial thickness at swollen regions was determined by plotting the signal intensity along the ROIs and measuring the gap between the two peak intensity values. Asterisks indicate significant differences with respect to the wild-type strain as determined by Student’s T-test. White bars = 5 μm.

When imaging mitochondria in fission-mutant cells, we observed that in certain regions they appeared swollen compared to WT cells, characterized by regions of increased distance between the mitochondrial outer membrane layers (Figure 2(A)). In addition, while WT mitochondria were overall of similar width, mitochondria in the fission-mutants also displayed thinner regions in between the more swollen sections. We quantified the increase in mitochondrial width by doing a targeted selection of swollen regions for each of the mutants and comparing their mitochondrial width to those of the more regular WT width. We find that the mitochondrial width in these swollen regions is at least two-fold higher in the mutants as compared to the WT (Figure 2(B)). The data confirm that mitochondrial fission plays a vital role in maintaining normal mitochondrial morphology in *A. nidulans*.

### Mitochondria exhibit a “beads on a string” phenotype in response to induced oxidative stress without the mitochondrial fission machinery

**Figure 3.**
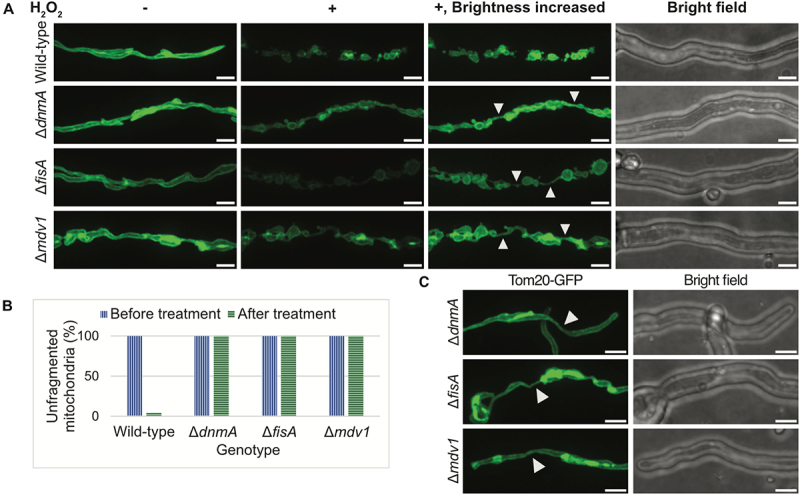
Deletion of genes encoding core components of the *A. nidulans* mitochondrial fission machinery results in inability to complete oxidative stress-induced mitochondrial fragmentation. (A) Spores from wild-type as well as the mitochondrial fission mutants were inoculated in minimal media and allowed to grow at 25°C for 18 h. Mitochondrial morphology was recorded before (left panel) and 20 min after treatment with 5 mM H_2_O_2_ (middle panels) using spinning-disc confocal microscopy. White arrowheads point to thin thread-like sections of mitochondria that connect the swollen sections. (B) Percentage of cells with unfragmented mitochondria before and 20 min after treatment with 5 mM H_2_O_2_. Data represents cells from at least 20 fields of view for each strain. (C) Sporadic thin thread-like sections of mitochondria are observed in the fission-mutant cells even in the absence of external perturbations, indicating sites where failed mitochondrial fission events might have been initiated without additional oxidative stress. White bars = 5 μm.

Garrido-Bazán et al. previously reported that mitochondrial fragmentation in response to induced oxidative stress in *A. nidulans* is an active process mediated by the mitochondrial fission machinery^[37]^. Induced oxidative stress in *A*. *nidulans* causes DnmA oxidation at cysteine-450, resulting in the formation of higher-order DnmA oligomers that are recruited to the sites of mitochondrial fission in a FisA-dependent manner^[40]^. In the absence of DnmA or FisA, Garrido-Bazán et al. demonstrated that exposure to induced oxidative stress resulted in the formation of multiple mitochondrial constrictions that failed to reach completion of fission^[41]^. To confirm the fission-mutants generated in our lab show similar resistance to mitochondrial fragmentation in response to induced oxidative stress, we treated WT and fission-mutant cells with 5 mM hydrogen peroxide (H_2_O_2_) and imaged mitochondria 20 min after, as previously reported^[37,40,41]^. In response to induced oxidative stress, mitochondria were fragmented in the WT cells 20 min after the addition of 5 mM H_2_O_2_ (Figure 3(A,B)). However, in cells from the fission-mutant strains, mitochondria were characterized by multiple constrictions that failed to complete fission (Figure 3(A,B)), as has been previously reported^[41]^, resulting in a “beads on a string” phenotype characterized by swollen sections of mitochondria which remained connected via narrow thread-like sections of mitochondria (Figure 3(A,B)). Mirroring prior findings by Garrido-Bazán et al., such narrow thread-like sections were observed sporadically in the fission-mutant cells in the absence of induced oxidative stress, though much less abundant (Figure 3(C); also seen in Figure 2(A))^[41]^. This shows that while exposure to induced oxidative stress might promote mitochondrial fission, similar events occur without increased imposition of external oxidative stress, albeit at a much lower frequency. The potential mechanism resulting in this phenotype will be discussed further in the discussion. Overall, these results confirm that the mitochondrial fission machinery is essential for the successful completion of mitochondrial fragmentation in response to induced oxidative stress in *A. nidulans*.

### Mitochondrial fragmentation during biofilm formation is dependent on the mitochondrial fission machinery

**Figure 4.**
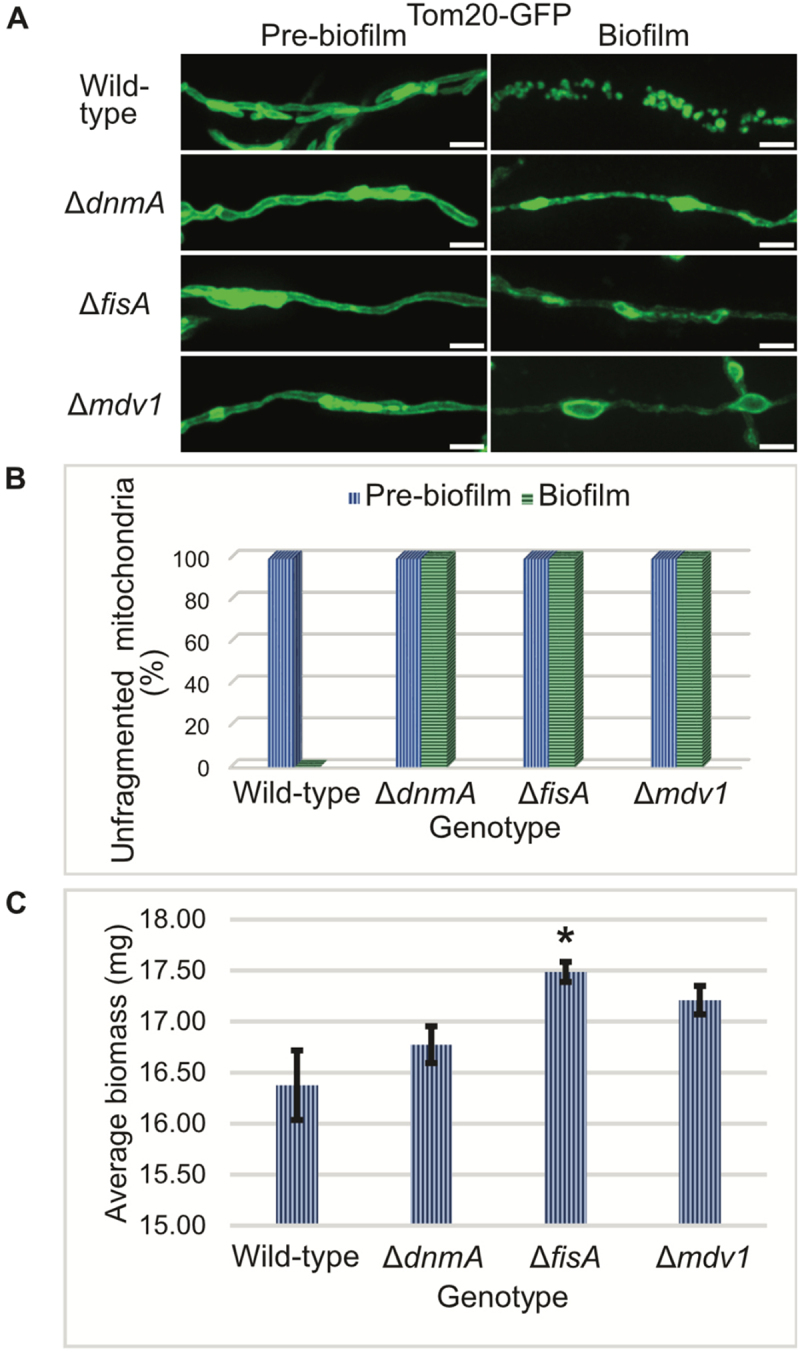
Deletion of genes encoding core components of the *A. nidulans* mitochondrial fission machinery results in an inability to undergo mitochondrial fragmentation during biofilm formation. (A) Spores from wild-type as well as the mitochondrial fission-mutants were inoculated in minimal media and allowed to grow at room temperature. Mitochondrial morphology was recorded before (left panel) and during biofilm formation (right panel) using spinning-disc confocal microscopy. (B) Percentage of cells with unfragmented mitochondria before and during biofilm formation. Data represents cells from at least 20 fields of view for each strain. (C) Spores from wild-type as well as the mitochondrial fission mutants were inoculated in minimal media and allowed to grow at room temperature for 5 days. Total biomass was harvested, dried, and dry-weight determined. Standard deviation from three biological replicates is indicated. Asterisks indicate significant differences with respect to the wild-type strain as determined by Student’s T-test. White bars = 5 μm.

To investigate the role of the mitochondrial fission machinery in mitochondrial fragmentation during biofilm formation (Figure 1), we imaged mitochondrial morphology in pre-biofilm and biofilm cells in both the WT as well as the fission-mutants. While mitochondria underwent fragmentation during biofilm formation in the WT cells, they remained tubular and unfragmented in the fission-mutants (Figure 4(A,B)), revealing complete mitochondrial fragmentation during biofilm formation is an active process mediated by the mitochondrial fission machinery. Mitochondria in the fission-mutant biofilm cells comprised of long narrow regions interspersed with swollen bead-like sections (Figure 4(A)), indicating mitochondrial fission is triggered in these cells during biofilm formation but fails to reach completion due to the absence of a functional mitochondrial fission machinery.

It is possible that the persistence of tubular unfragmented mitochondria in the fission-mutant biofilm cells might be caused by the growth inhibition of fission-mutants that might render them unable to reach a similarly advanced stage of biofilm growth as the WT cells at Day 3. To test this further, both the WT as well as the fission-mutant strains were allowed to grow for 5 days to form more mature biofilms, which were subsequently harvested, dried, and analyzed for biomass generation. Compared to the WT, a slight increase in biomass was observed for all the fission-mutants, but only the slight increase in biomass generation seen for Δ*fisA* cells was significant (Figure 4(C)). The data suggest that the persistence of tubular unfragmented mitochondria in fission-mutants is not caused by failure of the mutants to form mature biofilms. Notably, it has previously been noted that deletion of DnmA in *A. nidulans* led to growth inhibition on agar plates without affecting the biomass generation in submerged, but rotated, cultures^[39]^ and our findings indicate that a similar effect is seen without active media mixing.

### Microtubules (MTs) disassembly during biofilm formation is unperturbed by loss of biofilm mitochondrial fragmentation

**Figure 5.**
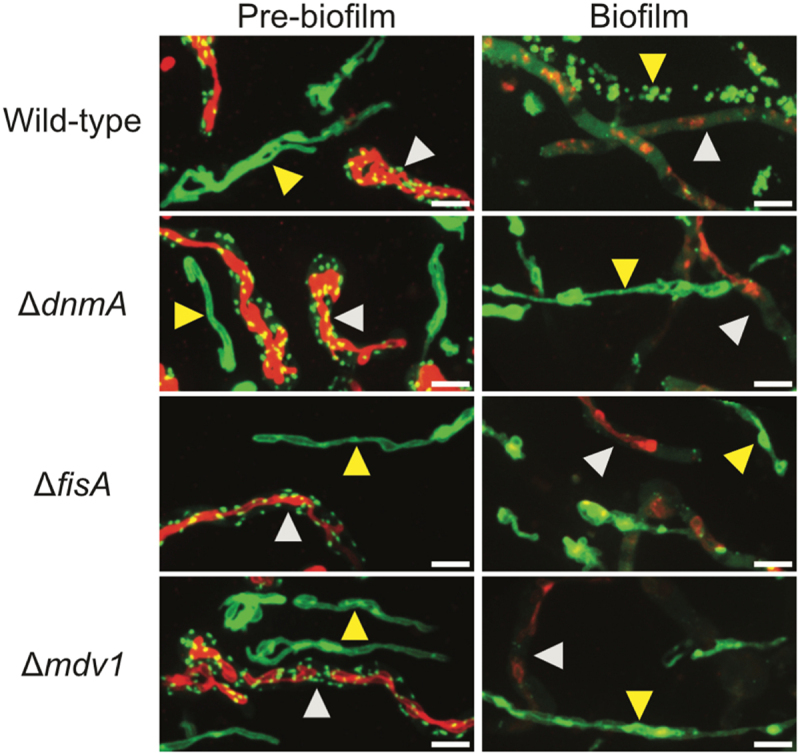
Microtubules dispersal during *A. nidulans* biofilm formation is unperturbed by the loss of biofilm mitochondrial fragmentation. Spores from strains expressing the mitochondrial tag Tom20-GFP (yellow arrowheads) were cocultured in a 50:50 ratio with spores from strains expressing both the MTs tag EB1-GFP and mitochondrial tag Tom20-mRFP (white arrowheads), for both wild-type as well as the fission-mutants as indicated. MT dynamics and mitochondrial morphology were tracked simultaneously before (left panel) and during biofilm formation (right panel) using spinning-disc confocal microscopy. White bars = 5 μm.

The disassembly of MTs as a consequence of biofilm formation has previously been reported by our lab^[22]^ and can be used as a marker for biofilm formation^[23]^. The protein EB1 (end-binding protein 1) localizes to the actively growing ends of MTs, and we have previously used EB1-GFP comets to track active MTs during biofilm formation^[22,23]^. Active MT growth relies on a continuous supply of energy in the form of GTP, and the disassembly of MTs during biofilm formation could potentially be affected by the loss of mitochondrial energy generation caused by biofilm mitochondrial fragmentation. To determine if mitochondrial fragmentation might be involved in driving the disassembly of MTs during biofilm formation, we tracked MT dynamics in both the WT as well as the fission-mutants during biofilm formation. To simultaneously track both these organelles using GFP-tagged markers, we co-cultured cells harboring the mitochondrial tag Tom20-GFP with cells harboring the MT tag EB1-GFP in a 50:50 ratio, for both the WT and the fission-mutants. We observed that during biofilm formation, MTs are dispersed in both the WT and the fission-mutant biofilm cells, even though the mitochondria remained unfragmented in the fission-mutant biofilm cells (Figure 5). These results indicate that mitochondrial fragmentation appears not to be required for MTs to be disassembled during biofilm formation. These results also indicate that even though the mitochondrial fission-mutants grow poorly on agar plates, the persistence of tubular unfragmented mitochondria in fission-mutant biofilm cells is not caused by failure of the mutants to form a biofilm because MT disassembly still occurs.

### Mitophagy is induced during biofilm formation in nitrogen-limiting conditions

**Figure 6.**
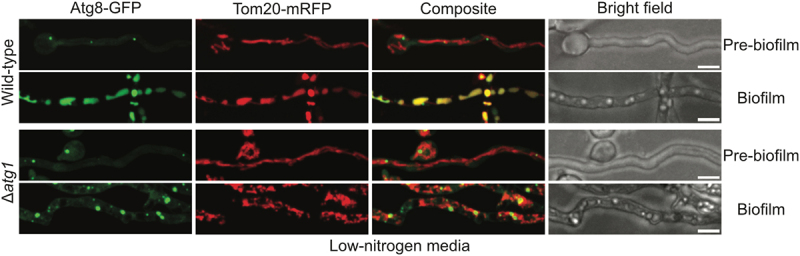
Mitophagy is induced during *A. nidulans* biofilm formation in nitrogen-limiting conditions. Spores from a strain expressing the autophagy marker GFP-Atg8 along with the mitochondrial outer membrane marker Tom20-mRFP were inoculated in nitrogen-limiting media and allowed to grow at room temperature. Autophagy dynamics and mitochondrial morphology were tracked simultaneously before and during biofilm formation using spinning-disc confocal microscopy. As a control, an autophagy-mutant strain deleted for the essential autophagy protein kinase Atg1 was similarly imaged. White bars = 5 μm.

Using the dynamic locations of the autophagy protein GFP-Atg8 (autophagy related protein 8) as a marker for autophagy, our lab has previously reported that autophagy is induced during biofilm formation in nutrient-limiting conditions with 1/20^th^ regular nitrogen concentration and 1/10^th^ regular carbon concentration^[23]^. Notably, autophagy was not observed during biofilm formation in standard minimal media with normal levels of nitrogen (10 mM ammonium tartrate) and carbon (55.5 mM glucose). Before biofilm formation, GFP-Atg8 locates to foci representing phagophore assembly sites (PAS) prior to autophagy (Figure 6, WT Pre-biofilm). When autophagy is induced during biofilm formation in nutrient-limiting conditions, GFP-Atg8 is processed to locate inside vacuoles where the GFP moiety accumulates due to its resistance against vacuolar proteases (Figure 6, WT Biofilm). Thus, accumulation of the GFP signal from GFP-Atg8 in vacuoles acts as a marker for autophagy^[23,42]^.

In pre-biofilm cells, mitochondria exist in a long tubular state while GFP-Atg8 located to PAS as expected (Figure 6, WT Pre-biofilm). In founder biofilm cells grown in nitrogen-limiting conditions, GFP-Atg8 located inside vacuoles, showing that autophagy is induced in these cells (Figure 6, WT Biofilm). Autophagic vacuoles in nitrogen-limited founder biofilm cells exhibited an elongated morphology of varying sizes (Figure 6, WT Biofilm and Table 1). Importantly, signals from the mitochondrial marker Tom20-mRFP were also found to locate inside vacuole-like structures that colocalized with vacuoles containing the autophagy-processed GFP-Atg8 signal, indicating mitochondria are targeted for degradation through mitophagy in these biofilm cells (Figure 6, WT Biofilm). We analyzed the colocalization of the mitochondrial Tom20-mRFP signal with the autophagy-processed GFP-Atg8 signal at the level of individual z-slices, confirming mitochondrial signals located inside the autophagic vacuoles in these biofilm founder cells (Figure S2).

**Table 1.** The effects of different nutritional conditions on mitochondrial dynamics and morphology in *Aspergillus nidulans* biofilm founder cells. *N* = nitrogen, C = carbon.

	N	C	Mitochondrial appearance	Autophagy	Mitophagy	Morphology of autophagic vacuoles
Wild-type	High	High	Fragmented	No	No	N/A
	Low	High	Unfragmented	Yes	Yes	Elongated
	High	Low	Fragmented	Yes	No	Spherical
Mitochondrial fission mutants	High	High	Unfragmented	No	No	N/A
	Low	High	Unfragmented	Yes	Yes	Elongated
	High	Low	Unfragmented	Yes	No	Spherical

To confirm the accumulation of GFP-Atg8 signal within vacuoles in founder biofilm cells is a result of mitophagy during biofilm development in nitrogen-limiting media, we tracked mitochondrial morphology and autophagy under similar conditions in a strain lacking the essential autophagy protein kinase Atg1. In the absence of Atg1, GFP-Atg8 remained in PAS in founder biofilm cells in nitrogen-limiting media (Figure 6, Δ*atg1* Biofilm), confirming the accumulation of GFP-Atg8 signal in vacuoles that colocalized with mitochondrial Tom20-mRFP signal in founder biofilm cells to be a result of mitophagy during biofilm development in nitrogen-limiting media.

### Mitophagy is independent of mitochondrial fission during biofilm formation in nitrogen-limiting conditions

Dnm1 is required for mitochondrial fission and was also identified in a screen for budding yeast mutants defective in mitophagy upon shifting to nitrogen-depleted media^[43]^. In addition, the interaction between Dnm1 and the autophagy protein Atg11 was found to be essential for efficient yeast mitophagy upon nitrogen starvation^[44]^. Similarly, in the insect pathogenic fungus *Beauveria bassiana*, deletion of either one of the three core components of the mitochondrial fission machinery resulted in loss of mitochondrial fragmentation and also blocked mitophagy upon mild starvation stress^[45]^. These findings suggest mitochondrial fission is essential for mitophagy in response to nitrogen starvation in various fungi.

**Figure 7.**
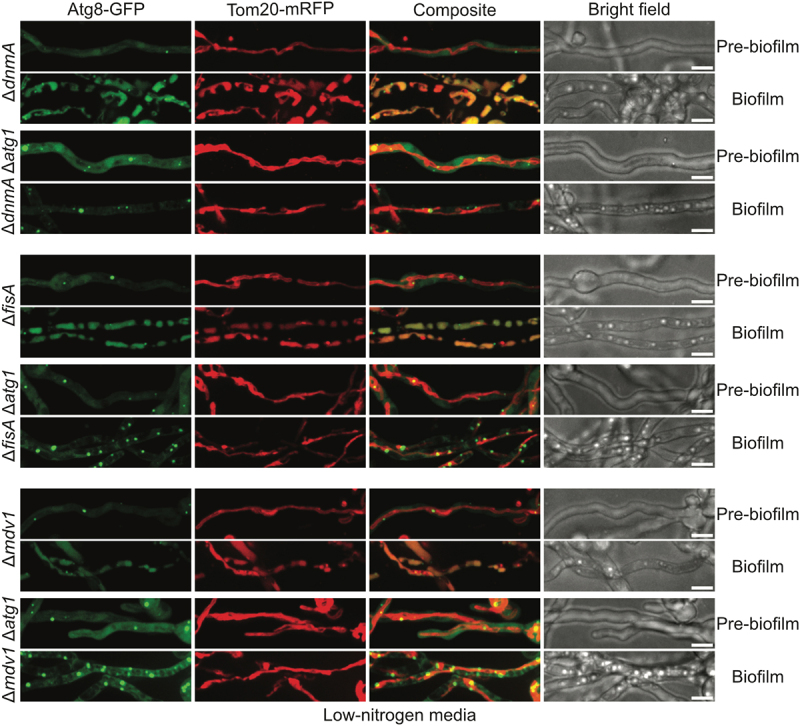
Mitophagy during *A. nidulans* biofilm formation in nitrogen-limiting conditions is independent of biofilm mitochondrial fragmentation. Spores from fission-mutant strains expressing the autophagy marker GFP-Atg8 along with the mitochondrial outer membrane marker Tom20-mRFP were inoculated in nitrogen-limiting media and allowed to grow at room temperature. Autophagy dynamics and mitochondrial morphology were tracked simultaneously before and during biofilm formation using spinning-disc confocal microscopy. As a control, corresponding autophagy-defective strains deleted for the autophagy protein kinase Atg1 were similarly imaged. White bars = 5 μm.

To investigate if mitochondrial fission is similarly required for *A. nidulans* mitophagy during biofilm formation in nitrogen-limiting conditions, we tracked the fate of mitochondria in the fission-mutants during biofilm formation in nitrogen-limiting conditions. In fission-mutant pre-biofilm cells, no mitophagy or autophagy was apparent, and mitochondria existed in a tubular unfragmented state while GFP-Atg8 located to PAS (Figure 7). After allowing biofilm formation, in the fission-mutant founder biofilm cells, the mitochondrial Tom20-mRFP signal located to vacuoles that colocalized with the autophagy-processed GFP-Atg8 signal (Figure 7) revealing activation of autophagy and mitophagy. The autophagic vacuoles observed in fission-mutant founder biofilm cells under nitrogen-limiting conditions resembled the variably sized elongated autophagic vacuoles observed in WT cells under similar growth conditions (Figure 7 and Table 1). This effect was observed in all the biofilm cells imaged from both the WT and the fission-mutants, indicating mitochondrial fission is not essential for *A. nidulans* mitophagy during biofilm formation in nitrogen-limiting conditions.

As a control, corresponding autophagy defective strains deleted for the autophagy protein kinase Atg1 were imaged in parallel, where GFP-Atg8 remained in PAS and mitochondria remained unfragmented in founder biofilm cells (Figure 7). The data confirm the accumulation of GFP-Atg8 and Tom20-mRFP signals in vacuoles results from autophagy/mitophagy during biofilm development in nitrogen-limiting media.

### Mitophagy does not occur during biofilm formation in carbon-limiting conditions although autophagy does

In budding yeast, both autophagy and mitophagy have been reported at different stages of growth in carbon-limiting batch cultures^[46]^. When grown in carbon-limiting media (0.2% w/v glucose instead of 2% w/v glucose), *S*. *cerevisiae* exhibits diauxic growth. Initially, in the glucose-utilizing phase, cells grow logarithmically, producing ethanol through glucose fermentation until glucose is depleted, at which point growth stops temporarily. When growth restarts in the ethanol-utilizing phase, cells utilize ethanol to generate ATP through oxidative phosphorylation in the mitochondria until ethanol is depleted, causing a second growth arrest^[46]^. Autophagy is induced in the ethanol-utilizing phase, but mitochondria are excluded from autophagy in this phase of growth as they are essential for ATP production through oxidative phosphorylation. However, in the ethanol-depleted phase, both autophagy and mitophagy are observed^[46]^.

**Figure 8.**
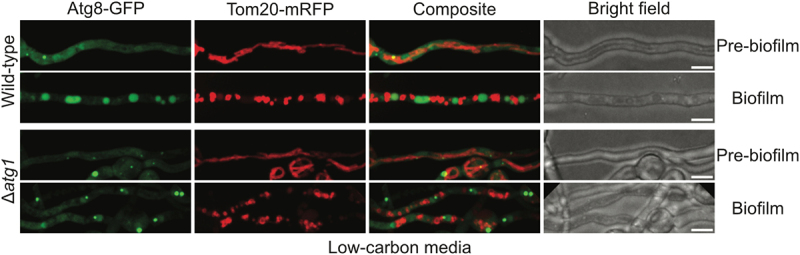
Mitophagy does not occur during *A. nidulans* biofilm formation in carbon-limiting conditions, even though autophagy is activated. Spores from a strain expressing the autophagy marker GFP-Atg8 along with the mitochondrial outer membrane marker Tom20-mRFP were inoculated in carbon-limiting media and allowed to grow at room temperature. Autophagy dynamics and mitochondrial morphology were tracked simultaneously before and during biofilm formation using spinning-disc confocal microscopy. As shown, autophagy was absent in an autophagy defective strain deleted for the autophagy protein kinase Atg1 under the same conditions. White bars = 5 μm.

We tested the effects of different concentrations of carbon on autophagy during biofilm formation and found that 0.55 mM glucose (1/100th regular carbon concentration) led to autophagy during biofilm development. We then tracked the fate of mitochondria during biofilm formation in carbon-limiting conditions. In pre-biofilm cells, mitochondria were in a tubular unfragmented state while GFP-Atg8 located to PAS as expected (Figure 8). In the founder biofilm cells, mitochondria had undergone fission into smaller fragments while the autophagy-processed GFP-Atg8 signal located in spherical vacuoles (Figure 8 and Table 1). Importantly, no discernible colocalization was observed between the mitochondrial Tom20-mRFP signal and the autophagic vacuoles containing the processed GFP signal. This indicates mitochondria are excluded from autophagic degradation during biofilm formation in carbon-limiting conditions (Figure 8) even though autophagy is active. Localization of the GFP-Atg8 signal in vacuoles was lost in Atg1-mutant founder biofilm cells (Figure 8), showing vacuolar GFP-Atg8 localization in WT cells during biofilm formation in carbon-limiting conditions is caused by autophagy.

Similar results were observed in the mitochondrial fission-mutant strains, where mitochondria remained unfragmented in founder biofilm cells and were excluded from autophagic degradation during biofilm formation in carbon-limiting conditions, as seen by the lack of any discernible colocalization of Tom20-mRFP signal with vacuoles containing the autophagy-processed GFP-Atg8 signal (Figure S3).

## Discussion

Our results using the model filamentous fungus *A*. *nidulans* show that mitochondria undergo mitophagy during biofilm formation under nitrogen-limiting growth but not when carbon is limited. This indicates that mitophagy is not a general response to biofilm formation but seems specific to biofilm development in nitrogen-limiting culture conditions. We find that biofilm formation results in a strong shift of mitochondrial dynamics toward fission, resulting in highly fragmented mitochondria in the founder biofilm cells. However, loss of mitochondrial fragmentation neither affected the ability to form a biofilm nor impeded mitophagy, indicating mitophagy need not depend on mitochondrial fragmentation to be successful. Table 1 summarizes the effects of different nutritional conditions on mitochondrial dynamics and morphology during biofilm formation, as defined in this study.

### Mitochondrial fragmentation in founder biofilm cells might be driven by increased reactive oxygen species (ROS) under hypoxia

Fungal biofilm development is associated with intrinsic development of self-generated hypoxia^[21]^, resulting from oxygen (O_2_) consumption during biofilm growth coupled with the inability of sufficient atmospheric O_2_ to penetrate into the inner biofilm layers. As the predominant consumers of O_2_ in cells, mitochondria are sensitive to changes in O_2_ availability in the microenvironment. Previous studies in both fungi and mammalian cell cultures have demonstrated increased ROS levels upon exposure to artificially imposed hypoxia^[47–51]^. Notably, the increased hypoxic ROS levels induced mitochondrial fragmentation in multiple mammalian systems^[52,53]^. Mitochondrial fragmentation observed in founder biofilm cells in this study could similarly be caused by self-generated biofilm hypoxia-induced increase in ROS levels. In support of this hypothesis, mitochondrial fragmentation occurs in non-biofilm cells in response to synthetic addition of ROS (5 mM H_2_O_2_) to the growth media, as has been previously reported^[37,40,41]^.

### Mitochondrial fragmentation in founder biofilm cells might reflect entry into a quiescent non-growing state

Mitochondria are dynamic organelles undergoing constant fusion and fission events that shape the mitochondrial morphology in response to changing physiological conditions. Tubular mitochondria are associated with active growing cells where they are involved in the generation of energy in the form of ATP^[54]^. In contrast, fragmented mitochondria are indicative of a quiescent state where they might not be actively generating ATP^[55]^. Prior studies have reported a marked reduction in mitochondrial processes including glycolysis and the TCA cycle in biofilm persister cells, suggesting mitochondrial function is downregulated in these cells^[25]^. In *C. albicans*, it has been reported that as biofilms developed, the levels of TCA cycle metabolites like succinate, fumarate, citrate, and malate are reduced in comparison to planktonic cells^[56]^. The dramatic shift in mitochondrial morphology toward a fragmented state during biofilm formation suggests mitochondrial function decreases in these cells, and they might be entering a dormant state of suspended growth.

### Swollen mitochondria in fission-mutants might represent ROS-induced mitochondrial uncoupling

Respiration, or the generation of ATP in mitochondria through oxidative phosphorylation, relies on the mitochondrial inner membrane potential and results in the generation of basal levels of ROS as a byproduct^[57]^. When cells experience oxidative stress caused by increased ROS levels, mitochondrial uncoupling acts as a defense mechanism to reduce the inner membrane potential, thereby protecting against further ROS generation by reducing the rate of respiration^[58]^. Garrido-Bazán et al. previously reported increased ROS in the *A. nidulans* mitochondrial fission-mutants and an associated decrease in mitochondrial respiration by ~20%^[37]^. Reduced membrane potential across the inner membrane has previously been reported to result in increased mitochondrial volume in rat neurons^[59]^. Taken together, we propose that in the fission-mutants, increased generation of mitochondrial ROS results in mitochondrial uncoupling and proton leakage as a defense mechanism, which in turn leads to weakening of the inner membrane potential causing increased mitochondrial volume and a swollen appearance as we have observed.

### “Beads on a string” mitochondrial phenotype and the mitochondria-endoplasmic-reticulum (ER) contact sites (MERCS)

Mitochondria and the ER are physically tethered at mitochondria-endoplasmic reticulum contact sites, abbreviated MERCS, via the ER-mitochondria encounter structure (ERMES) complex^[60]^. The ERMES complex in budding yeast comprises the four core proteins Mmm1, Mdm10, Mdm12, and Mdm34 and plays a crucial role in shaping the mitochondrial morphology^[61]^. MdmB, a subunit of ERMES complex in *A*. *nidulans* and the homolog of budding yeast Mdm10, is essential for mitochondrial constriction and fission in response to induced oxidative stress^[41]^. Time-lapse imaging has unveiled that mitochondria are constricted by ER tubules at contact sites between mitochondria and the ER prior to fission in budding yeast as well as in mammalian Cos-7 cells^[62]^. The sites of mitochondrial constriction in response to induced oxidative stress in *A*. *nidulans* were encompassed by or in contact with endoplasmic reticulum tubules^[41]^. These findings indicate a model where prior constriction by ER tubules at the sites of fission precedes actual mitochondrial fission. This model is supported by the fact that the helices formed by Dnm1 polymerization in budding yeast are much smaller in size compared to the diameter of mitochondria, thus necessitating prior constriction by ER tubules to enable the subsequent recruitment of Dnm1 helices at the sites of fission^[62]^.

The appearance of “beads on a string” phenotype in the fission-mutants upon induced oxidative stress suggests that mitochondrial constriction by ER tubules is initiated in these cells, leading to the appearance of narrow constricted thread-like sections of mitochondria. However, failure to complete subsequent fission in the absence of one or more core components of the mitochondrial fission machinery could result in the swollen sections of mitochondria remaining connected via the ER-constricted, narrow thread-like sections of mitochondria, giving rise to the appearance of “beads on a string” phenotype. These findings suggest that MERCS, formed by the ERMES complex, may have a role to play in regulating mitochondrial morphology during fungal biofilm formation.

### Nutrient availability during A. nidulans biofilm formation determines cellular targets for biofilm autophagy

Biofilm formation is not followed by mitophagy and/or autophagy under conditions of high carbon and nitrogen availability, suggesting these processes to be triggered only under specific nutritional cues. Our lab has previously reported autophagy occurs during biofilm development in nutrient-limiting media^[23]^. In this study, we have selectively varied the availability of carbon or nitrogen, further dissecting the cellular biology of biofilm development under specific nutrient-limiting conditions. We report that during biofilm formation, mitophagy is induced in nitrogen-limiting conditions but not in carbon-limiting conditions, even though autophagy is triggered under carbon-limiting conditions. In nitrogen-limiting media, where availability of glucose is maintained, the cells might be able to utilize energy generated through glycolysis and fermentation, rendering mitochondrial respiration unessential and therefore mitochondrial components available to recycling through mitophagy. However, in carbon-limiting media where fermentable sugars might reach depletion during biofilm development, cells might be forced to rely on mitochondrial respiration for energy generation, rendering mitochondria essential and protected from mitophagy. These findings demonstrate that the cell biology of biofilm cells can be variable and adaptable to different conditions in their microenvironments.

### Biofilm mitophagy in nitrogen-limiting conditions is independent of biofilm mitochondrial fragmentation

Mitophagy is the selective removal of mitochondria through autophagy. Mitophagy acts to regulate mitochondrial abundance depending on the cellular metabolic demand, and to perform quality control by eliminating damaged mitochondria. Multiple prior studies in budding yeast and filamentous fungi have demonstrated that core components of the mitochondrial fission machinery are essential for efficient induction of mitophagy upon shifting cells to nitrogen-depleted media^[43–45]^. The requirement of mitochondrial fission for mitophagy in budding yeast is believed to stem from the fact that on average, mitochondria are much larger in size than the autophagosomes that engulf them and hence need to be fragmented prior to mitophagy. Another hypothesis suggests that when damaged mitochondria are marked for mitophagy, mitochondrial fission segregates the damaged mitochondria from the healthy mitochondria to enable selective degradation of only the damaged mitochondria.

While most prior studies have used a shift from nitrogen-replete media to nitrogen-depleted media to induce mitophagy in budding yeast and other fungi, our results demonstrate the induction of mitophagy during fungal biofilm formation in nitrogen-limiting growth conditions. Importantly, mitophagy induction during biofilm formation in nitrogen-limiting conditions was not affected by the loss of mitochondrial fission, suggesting mitochondrial fission is not necessarily required for biofilm mitophagy. We found at least one other study in budding yeast where mitophagy induction was reported to be independent of mitochondrial fission, with the key difference being that mitophagy was induced through rapamycin treatment and not by shifting the cells to nitrogen-depleted media^[63]^. Rapamycin treatment is known to induce stress response resulting in growth arrest, which might mimic the conditions faced by founder biofilm cells as they enter a dormant state of suspended growth.

More recently, a study in HeLa cells reported that upon exposure to artificially imposed hypoxia, mitochondrial morphology shifted to large spherical units called megamitochondria, which engulfed lysosomes in a process termed “megamitochondria engulfing lysosome” (MMEL) to mediate mitochondrial self-digestion (MSD) by the lysosomal lytic enzymes^[64]^. Notably, MSD through MMEL did not require prior mitochondrial fission into tiny fragments, supporting a model where mitochondrial degradation in response to hypoxia is not dependent on mitochondrial fission.

The contradiction of mitochondrial fission being essential for mitophagy in some studies while being dispensable in others, including ours, could stem from the differences in the conditions used to induce mitophagy. How mitophagy is initiated or regulated in response to different stimuli is far from being understood. Compared to reports where mitophagy was induced in response to shifting cells to nitrogen-depleted media, a key distinction in our study is that the founder biofilm cells carrying out mitophagy are experiencing biofilm-generated hypoxia as well as nitrogen limitation. Mitochondrial generation of harmful ROS is known to increase under hypoxia, a potentially deleterious event that could harm the cells^[47,49,65]^. Biofilm-generated hypoxia could similarly drive the generation of toxic levels of ROS in the mitochondria, causing the founder biofilm cells to induce mitophagy as a defense against ROS-induced damage. Further research is warranted to decipher why prior mitochondrial fission is essential for mitophagy only in certain conditions.

## Supplementary Material

Supplemental Material

Table S1.docx

## Data Availability

All datasets generated as a part of this study are included in the article/supplemental material.
