# Supplementary figures and images for: During Aspergillus nidulans nitrogen-limited biofilm formation, mitophagy is independent of mitochondrial fission
Source: Autophagy Rep. 2025 Aug 22;4(1):2547194. doi: 10.1080/27694127.2025.2547194 (PMC12377116; doi:10.1080/27694127.2025.2547194)

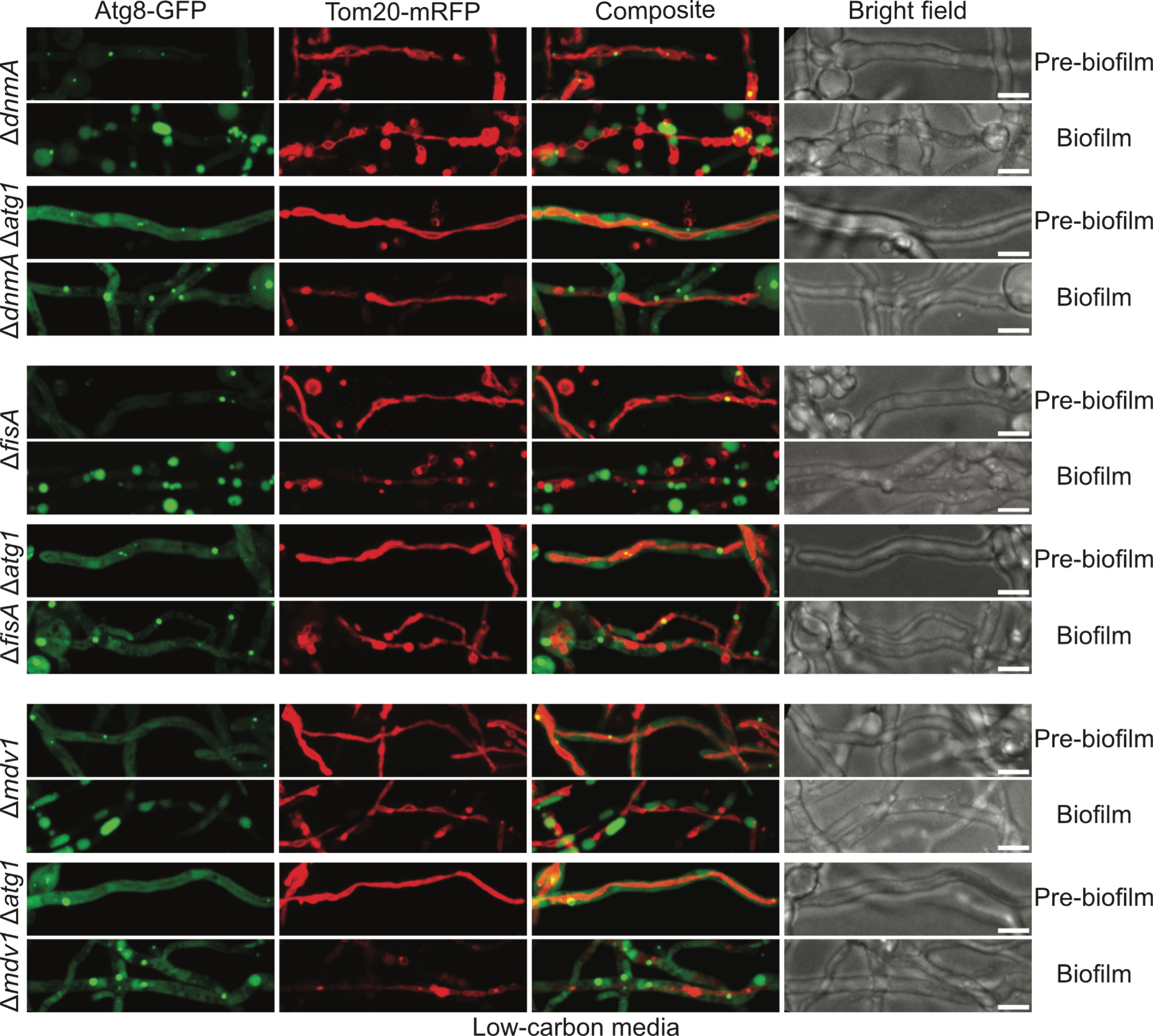

Supplement: Supplemental Material [file KAUO_A_2547194_SM4459.zip › Figure_S3.jpg]

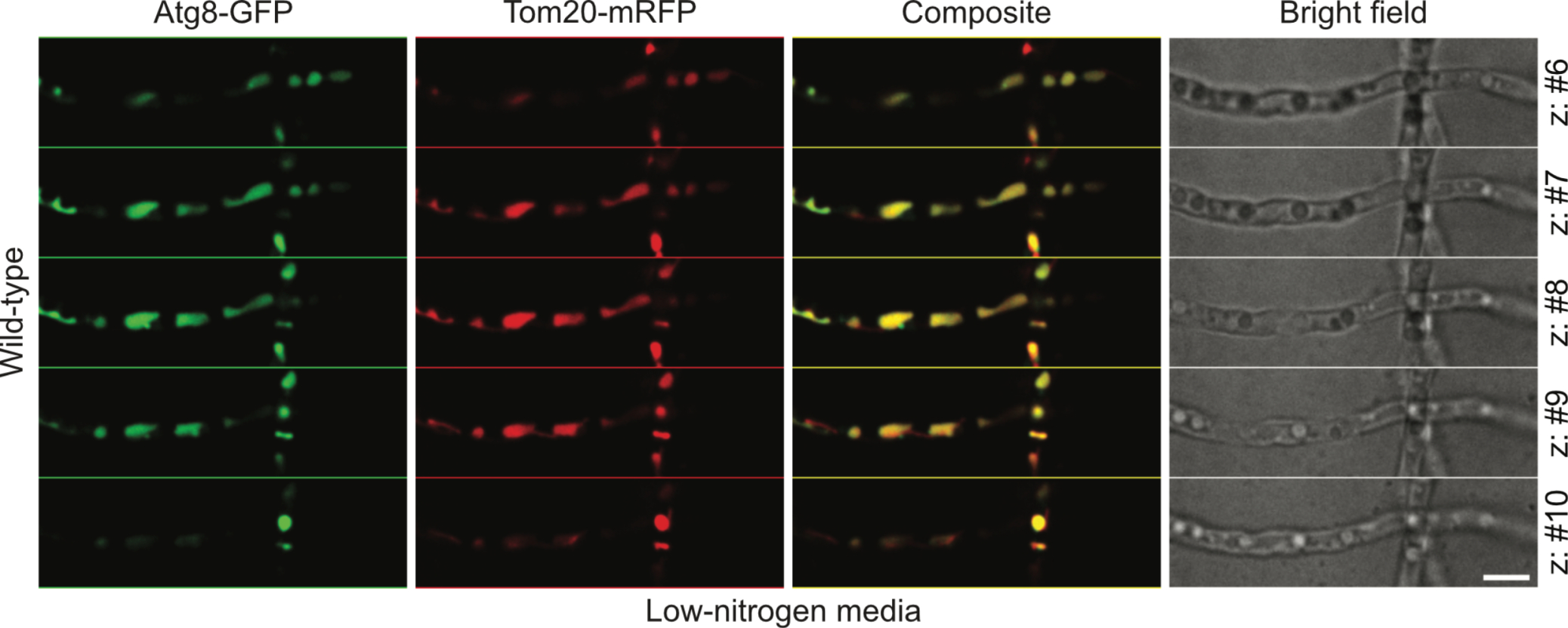

Supplement: Supplemental Material [file KAUO_A_2547194_SM4459.zip › Figure_S2.jpg]

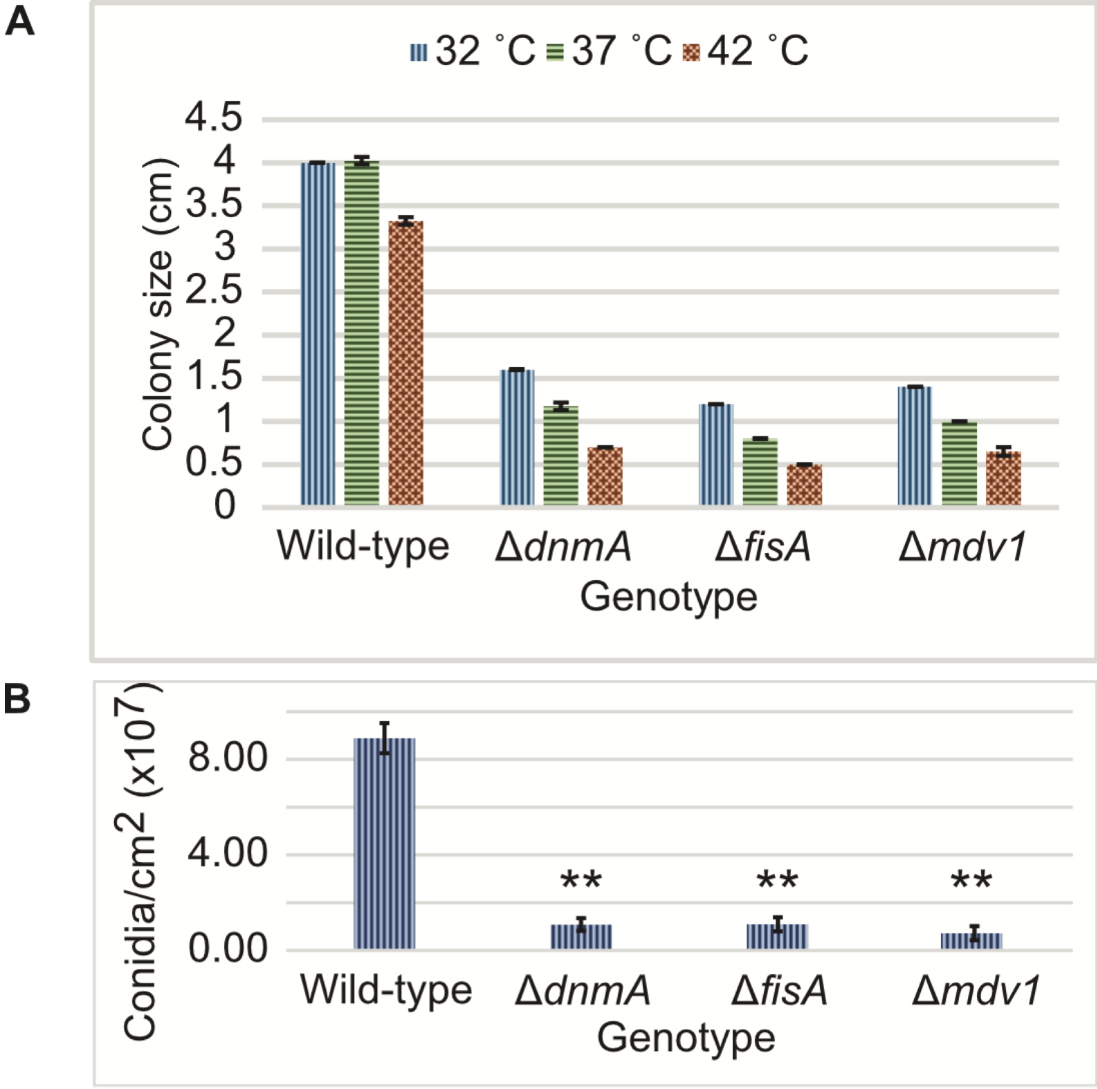

Supplement: Supplemental Material [file KAUO_A_2547194_SM4459.zip › Figure_S1.jpg]
