# Supplementary material for: During Aspergillus nidulans nitrogen-limited biofilm formation, mitophagy is independent of mitochondrial fission
Source: Autophagy Rep. 2025 Aug 22;4(1):2547194. doi: 10.1080/27694127.2025.2547194 (PMC12377116; doi:10.1080/27694127.2025.2547194)
Supplement: Table S1.docx [file KAUO_A_2547194_SM0542.docx]

**Table S1.** List of *A. nidulans* strains used in this study.

| **Strain** | **Genotype** |
| --- | --- |
| **SO1950** | *Tom20-GFP::pyrG^Af^*; *pyrG89*; *wA3*. |
| **HK194** | Δ*dnmA::pyro^Af^*;*Tom20-GFP::pyrG^Af^*; *pyroA4*; *pyrG89*; *wA3*. |
| **HK195** | Δ*fisA::pyro^Af^*;*Tom20-GFP::pyrG^Af^*; *pyroA4*; *pyrG89*; *wA3*. |
| **HK197** | Δ*mdv1::pyro^Af^*;*Tom20-GFP::pyrG^Af^*; *pyroA4*; *pyrG89*; *wA3*. |
| **SO1836** | *Tom20-mRFP::pyrG^Af^*; *EB1-GFP::pyro^Af^*; *pyrG89*; *pyroA4*; *wA3*. |
| **HK190** | Δ*dnmA::pyro^Af^*; *Tom20-mRFP::pyrG^Af^*; *EB1-GFP::pyrG^Af^*; Δ*nKuA::argB*; *pyroA4*; *pyrG89*; *argB2*. |
| **HK191** | Δ*fisA::pyro^Af^*; *Tom20-mRFP::pyrG^Af^*; *EB1-GFP::pyrG^Af^*; Δ*nKuA::argB*; *pyroA4*; *pyrG89*; *argB2*. |
| **HK193** | Δ*mdv1::pyro^Af^*; *Tom20-mRFP::pyrG^Af^*; *EB1-GFP::pyrG^Af^*; Δ*nKuA::argB*; *pyroA4*; *pyrG89*; *argB2*. |
| **HK179** | *Tom20-mRFP::pyrG^Af^*; *pyroA*-gpdAmini::GFP::atg8*; Δ*mus51::argB*; *pyrG89*; *pyroA4*; *argB2*. |
| **HK257** | Δ*atg1::pyrG^Af^*; *Tom20-mRFP::pyrG^Af^*; *pyroA*-gpdAmini::GFP::atg8*; *pyrG89*; *pyroA4*. |
| **HK175** | Δ*dnmA::pyro^Af^*; *pyroA*-gpdAmini::GFP::atg8*; *Tom20-mRFP::pyrG^Af^*; *pyroA4*; *pyrG89*. |
| **HK258** | Δ*atg1::pyrG^Af^*; Δ*dnmA::pyro^Af^*; *pyroA*-gpdAmini::GFP::atg8*; *Tom20-mRFP::pyrG^Af^*; *pyroA4*; *pyrG89*. |
| **HK176** | Δ*fisA::pyro^Af^*; *pyroA*-gpdAmini::GFP::atg8*; *Tom20-mRFP::pyrG^Af^*; *pyroA4*; *pyrG89*. |
| **HK259** | Δ*atg1::pyrG^Af^*; Δ*fisA::pyro^Af^*; *pyroA*-gpdAmini::GFP::atg8*; *Tom20-mRFP::pyrG^Af^*; *pyroA4*; *pyrG89*. |
| **HK178** | Δ*mdv1::pyro^Af^*; *pyroA*-gpdAmini::GFP::atg8*; *Tom20-mRFP::pyrG^Af^*; *pyroA4*; *pyrG89*. |
| **HK261** | Δ*atg1::pyrG^Af^*; Δ*mdv1::pyro^Af^*; *pyroA*-gpdAmini::GFP::atg8*; *Tom20-mRFP::pyrG^Af^*; *pyroA4*; *pyrG89*. |
